# Base of the Fifth Metatarsal: Fracture or Anomaly?

**DOI:** 10.7759/cureus.97610

**Published:** 2025-11-23

**Authors:** Leen Al Zayer, Abdulrahman Alrujaib, Ayleen Aamir, Ahmed Silman, Ahmed Siddiqui

**Affiliations:** 1 School of Medicine, Royal College of Surgeons in Ireland, Busaiteen, BHR; 2 Department of Orthopedic Surgery, Bahrain Defence Force Hospital, Riffa, BHR

**Keywords:** accessory ossicle, avulsion fracture, foot pain, misdiagnosis, os vesalianum pedis, young athlete

## Abstract

Accessory ossicles are anatomical variants that may lead to foot pain, particularly in individuals with a history of trauma, and are often overlooked. We present a case of a 21-year-old male patient with a history of inversion injuries who was initially misdiagnosed with an avulsion fracture of the fifth metatarsal. Following multiple interventions for the fracture, including surgical fixation with plates, it was later discovered that he had a bilateral os vesalianum pedis, which was identified as the source of his pain. This case emphasizes the importance of accurate diagnosis and management of accessory ossicles, particularly in young athletes, to avoid unnecessary treatments.

## Introduction

The foot and ankle are not spared from anatomic variations. An accessory ossicle is a well-ossified structure located proximal to a bone or joint that is congenital in origin and is a product of the incomplete fusion of ossification centers [1]. The prevalence of accessory ossicles of the foot can be as high as 26%. However, os vesalianum pedis (OVP) is notably rare, with a low prevalence of 0.1% [2]. OVP is a small accessory ossicle located proximally to the base of the fifth metatarsal at the insertion point of the peroneus brevis tendon [3]. Like other accessory ossicles, OVP is usually asymptomatic; however, in rare cases, it can present with lateral foot pain [1]. The pain, although poorly understood, has been attributed to direct trauma such as lateral foot contusions or ankle inversion sprains, or to repetitive microtrauma [4]. This paper presents an unusual occurrence of a previously asymptomatic OVP that developed symptoms following an injury involving inversion of the right foot to demonstrate the importance of accurate diagnosis and management of accessory ossicles to avoid unnecessary treatments.

## Case presentation

**Figure 1:**
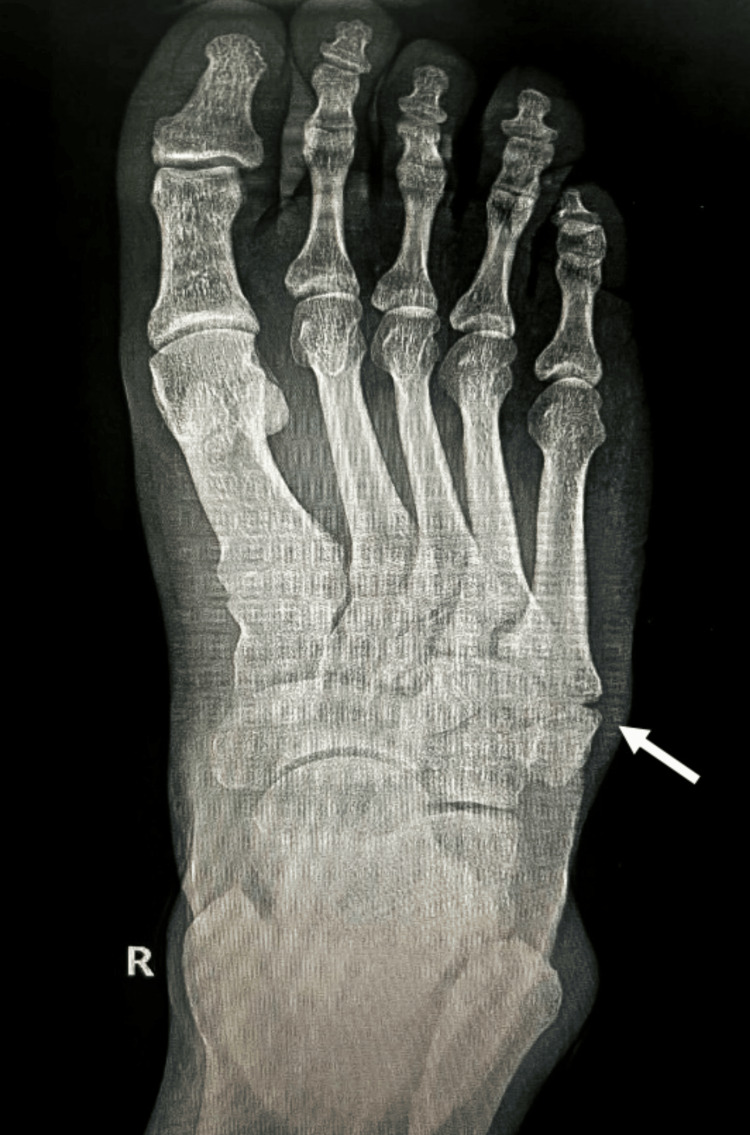
Initial X-ray of the right ankle following a twisting injury, demonstrating a fracture at the base of the fifth metatarsal

A 21-year-old male with a history of inversion injuries and no background of medical illness, family, or psychosocial history presented to the ED in 2018 after twisting his right ankle while playing football. He reported significant pain localized to the anterior talofibular ligament area, accompanied by marked edema that made shoe removal difficult. Radiographic evaluation revealed a fracture at the base of the fifth metatarsal, as seen in Figure 1. The injury was managed conservatively with a cast for three weeks, although the patient did not comply with the recommended use of an ankle boot walker.

**Figure 2:**
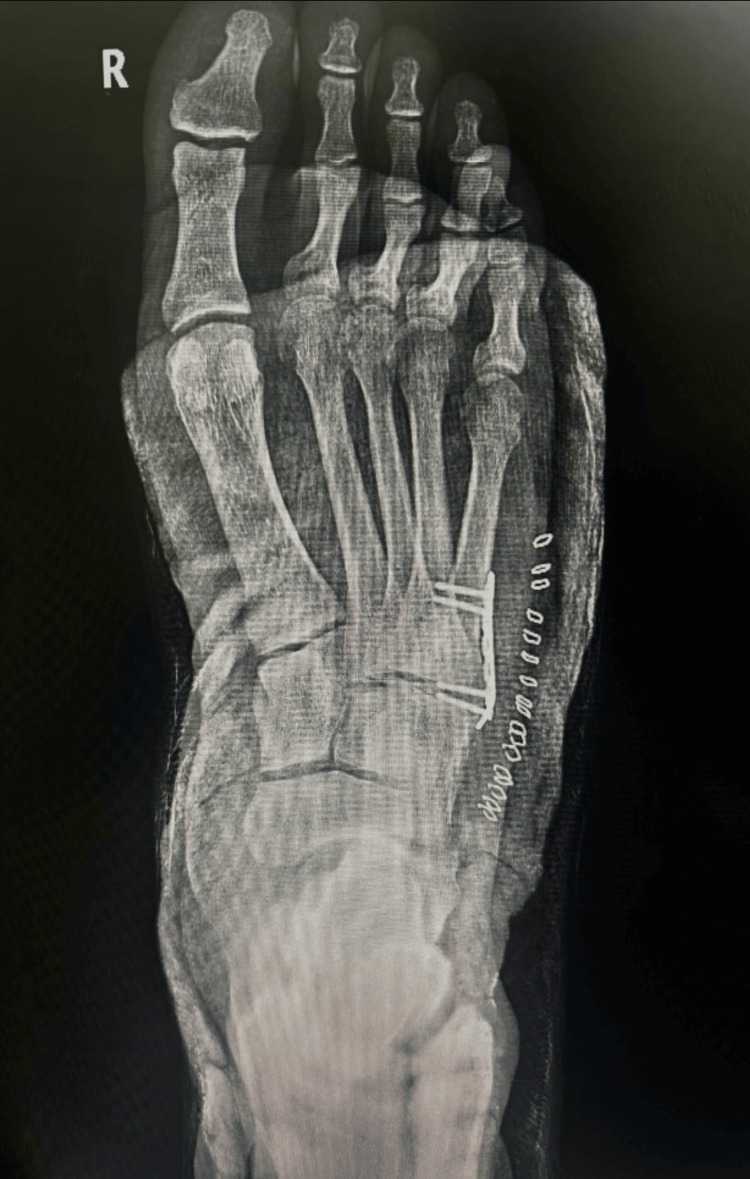
Postsurgical intervention to stabilize the fracture with plates

In 2022, the patient returned to the ED, reporting pain after an impact to the right foot while playing football. Imaging identified a nonunion of the previously fractured fifth metatarsal. The presence of fibrous tissue at the fracture site increased the risk of dislocation with further trauma. Following two weeks of persistent pain, the patient underwent surgical intervention to stabilize the fracture with plates, as shown in Figure 2. Notably, healing was suboptimal, with only 5-10% healing at three months and 20-30% at six months, prompting recommendations for non-weight-bearing for an additional four months.

**Figure 3:**
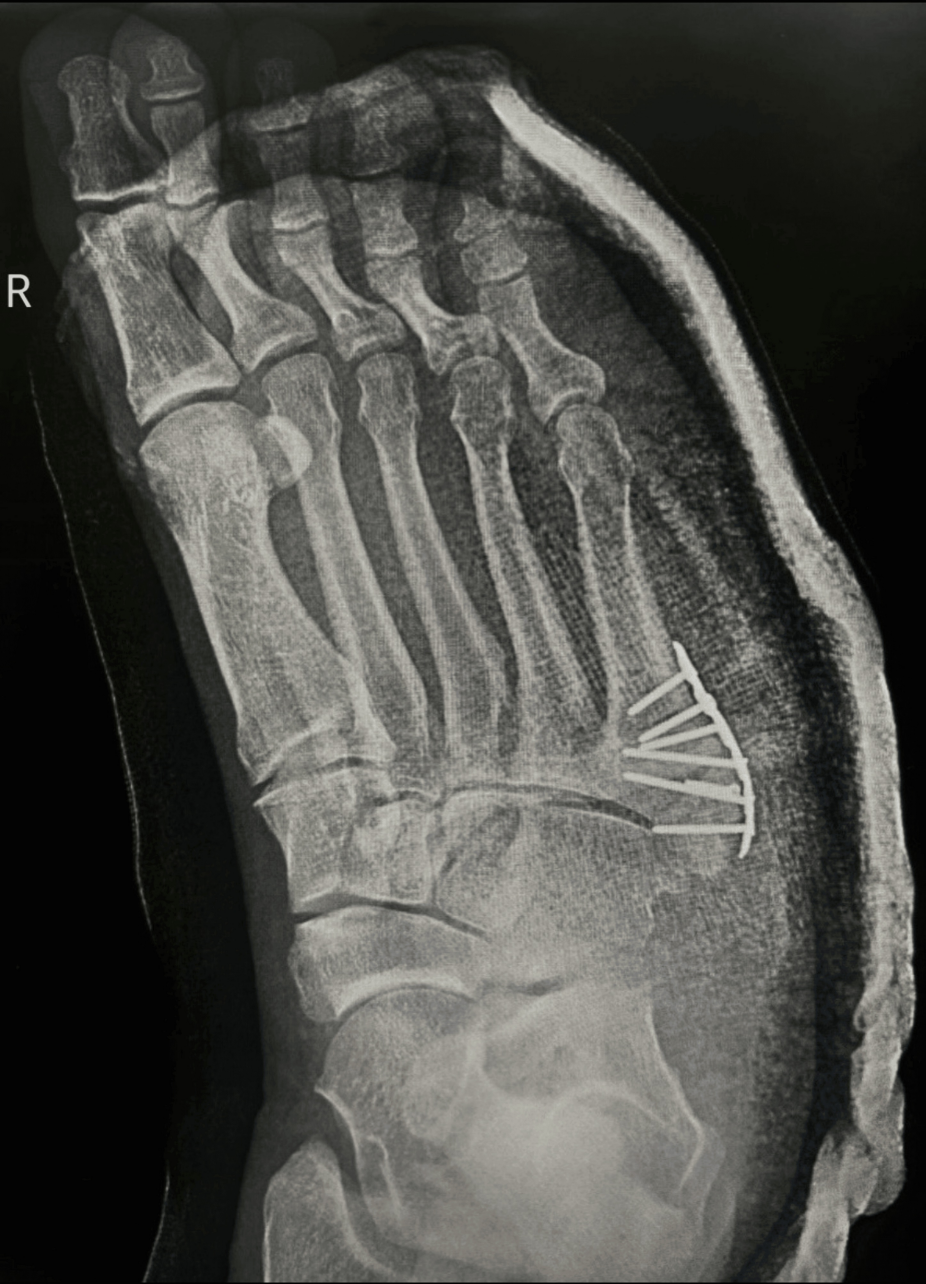
Right foot with an ankle boot walker after surgery to remove plates, debride the area, and insert screws

Seeking a second opinion, the patient underwent further surgery to remove the plates, debride the area, and insert screws, as shown in Figure 3. Despite these interventions, healing remained inadequate, leading to a change in management: removal of the ankle boot walker and encouragement to ambulate normally. This resulted in screw protrusion through the skin, which the patient self-managed by removing the screw, ultimately leading to the extraction of all remaining medical hardware.

**Figure 4:**
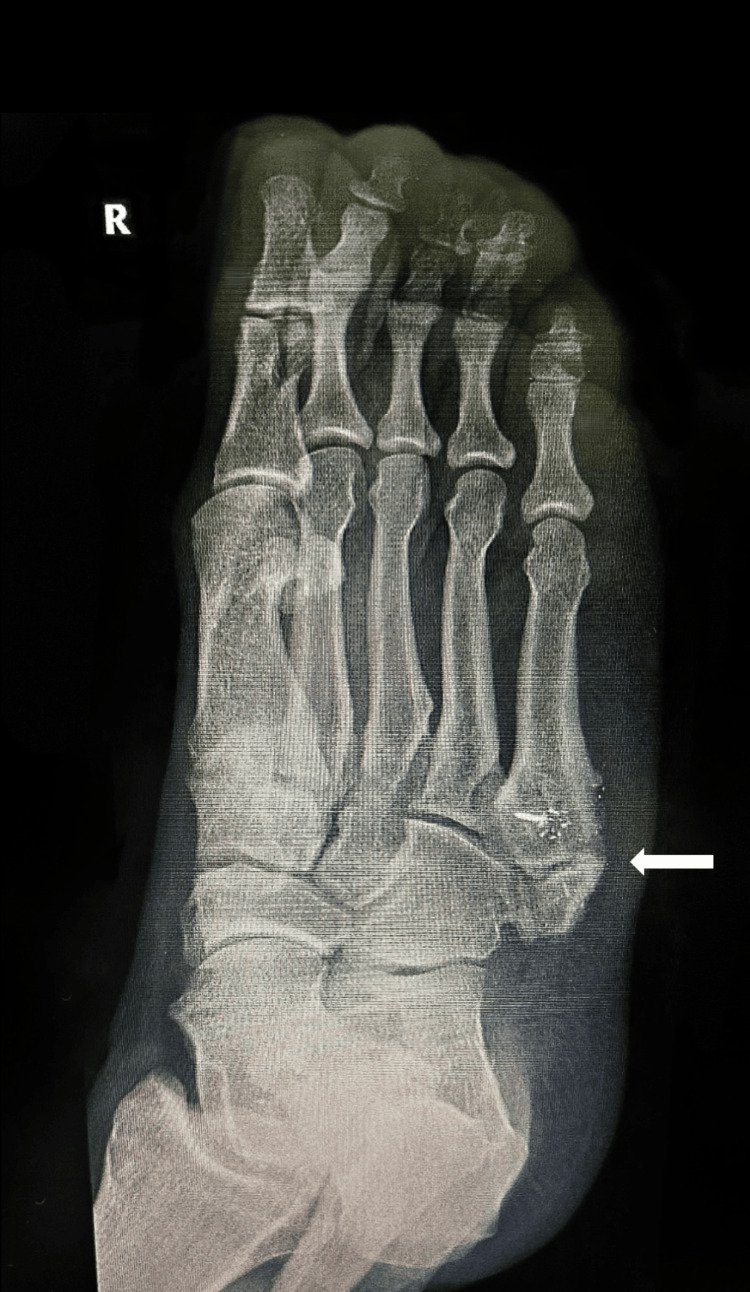
Ankle X-ray revealing the presence of an OVP bilaterally (right) OVP, os vesalianum pedis

**Figure 5:**
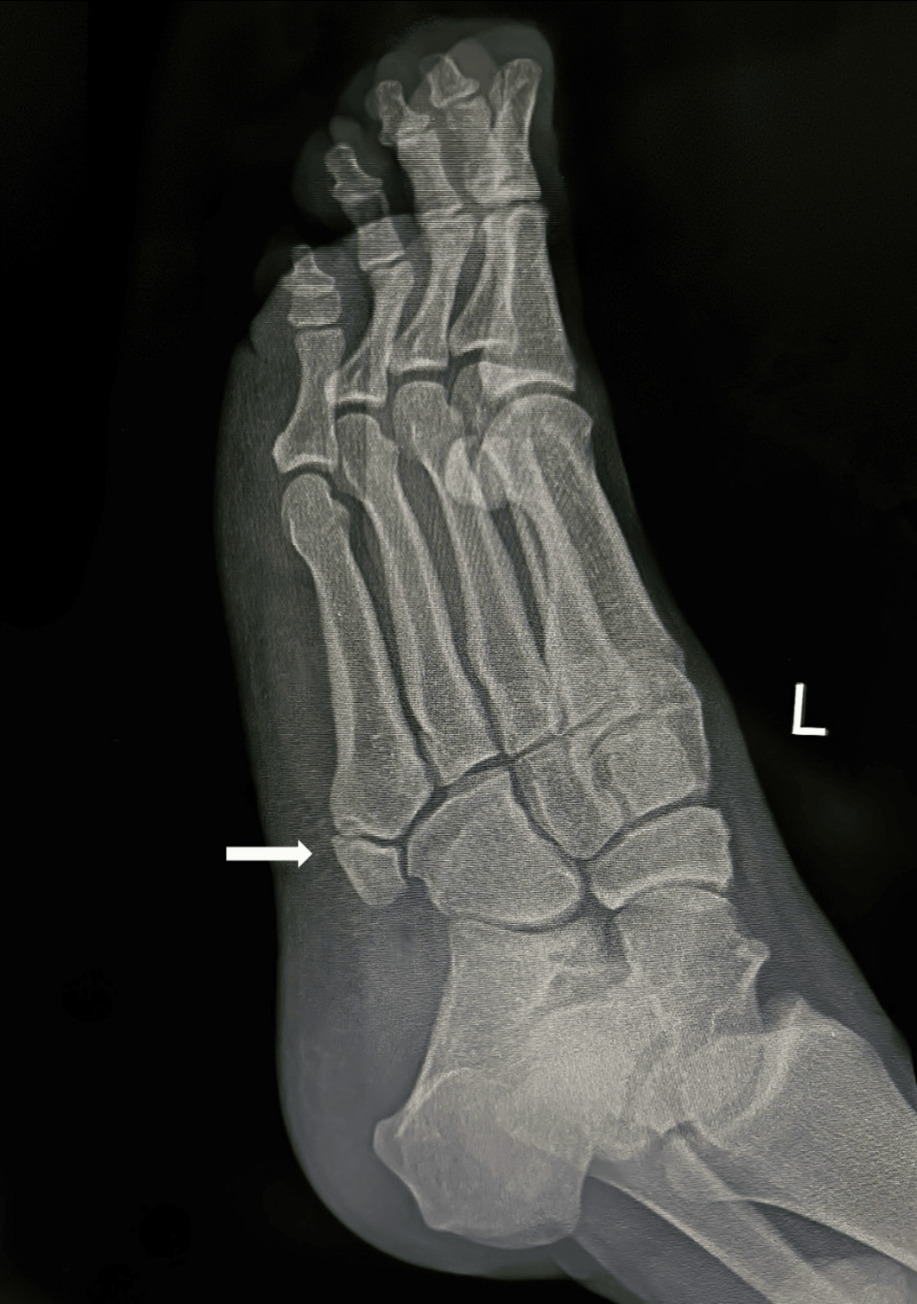
Ankle X-ray revealing the presence of an OVP bilaterally (left) OVP, os vesalianum pedis

In 2024, the patient began experiencing pulsating pain in the dorsal aspect of his left foot, described as “a hammer hitting his foot,” during a holiday. This pain improved with icing and analgesics. Subsequent X-ray imaging revealed the presence of an OVP bilaterally, which was determined to be the source of his exertional pain, as shown in Figure 4 and Figure 5.

This diagnosis indicated that no surgical intervention was necessary for this anatomical variant. At present, the patient was offered surgical excision of the OVP, but he opted for conservative management. No further follow-ups were required.

## Discussion

OVP is a rare accessory ossicle of the foot located proximal to the base of the fifth metatarsal, found within the tendon of the peroneus brevis and sometimes articulating with the cuboid [5]. It has a reported incidence of approximately 0.1-5.9% [6]. Accessory ossicles exhibit a wide range of morphological differences; thus, their exact prevalence remains unclear due to variations in study designs and participant demographics [7].

The misdiagnosis of an accessory ossicle as a fracture is a common clinical error with significant implications for patient management. They are often incidentally discovered on X-rays and may mimic fractures or loose bodies, creating a diagnostic dilemma. When the diagnosis is unclear, clinicians should maintain a high index of suspicion for accessory ossicles in patients presenting with foot pain. Orthopedic specialists may benefit from comparing radiographs of the affected foot with the contralateral foot to identify anatomical variations, followed by advanced imaging techniques such as computed tomography, magnetic resonance imaging, and bone scans to improve diagnostic accuracy [8]. Oblique radiographs of the foot are essential for visualizing the OVP [5], which is characterized by a well-defined corticated border, smooth rounded shape, and articulation with the cuboid bone. The absence of periosteal reaction and the presence of a consistent radiolucent space between the ossicle and the metatarsal further support the diagnosis of OVP and are key features distinguishing accessory ossicles from fractures [6,9].

While typically asymptomatic, accessory ossicles can become symptomatic, particularly in individuals with existing foot pathologies or those exposed to trauma or repetitive stress. This may result in pain or discomfort, particularly in the lateral foot in cases of OVP [10]. In this paper, the patient’s symptoms were initially misdiagnosed as an avulsion fracture of the fifth metatarsal, and he underwent inappropriate treatment with open reduction and internal fixation. However, the subsequent development of similar symptoms in the contralateral foot prompted further investigation, revealing a similar pattern on radiographs of the opposite foot. Similar cases have been reported in the literature, where young males presented to the ED with an acute ankle sprain and lateral foot pain, and subsequent radiographs revealed an accessory ossicle [9,10].

Given that most accessory ossicles are asymptomatic, treatment typically involves observation. However, if symptoms develop, management depends on the location of the ossicle and the chronicity of symptoms [11]. Conservative measures include limited weight-bearing, orthotics, targeted exercises, and nonsteroidal anti-inflammatory drugs [10]. If these measures are ineffective, surgical options such as osteosynthesis, bone grafting, or excision of the OVP without disrupting the peroneus brevis tendon insertion may be considered [9].

## Conclusions

This case report highlights the importance of accurate radiographic interpretation and a thorough understanding of anatomical variants, such as OVP, particularly in athletes with a history of trauma. It underscores the need for orthopedic specialists to maintain suspicion for anatomical variants when evaluating foot pain to prevent misdiagnosis and inappropriate or unnecessary treatment. Further research into the management of accessory ossicles and their implications for patient care is warranted to enhance diagnostic accuracy and treatment outcomes.
